# Psychometric examination of Runco Ideational Behavior Scale: Thai adaptation

**DOI:** 10.1186/s41155-020-00170-9

**Published:** 2021-01-11

**Authors:** Puthyrom Tep, Sorakrich Maneewan, Saranya Chuathong

**Affiliations:** 1 Learning Innovation and Technology Program, Faculty of Industrial Education and Technology, King Mongkut’s University of Technology Thonburi, Bangkok, Thailand; 2 Department of Educational Communications and Technology, Faculty of Industrial Education and Technology, King Mongkut’s University ofTechnology Thonburi, Bangkok, Thailand

**Keywords:** Creativity assessment, Ideational behavior, Psychometric examination, RIBS Thai version, Runco Ideational Behavior Scale, Undergraduate student

## Abstract

Creativity is a multidimensional construct. Several different approaches have been developed to measure creativity, including psychometric scales. The Runco Ideational Behavior Scale (RIBS) is one such measure of creative ideation. The primary purpose of this paper was to assess the 23 items of the RIBS in the context of the Thai language and examine scale reliability and validity. Participants, consisting of 508 undergraduate students selected from five Thai public universities, were selected through a convenience sampling approach involving both exploratory and confirmatory factor analysis. Results suggested that the Thai version of the RIBS presented a valid measure to a certain extent. Factor analysis of the empirical data indicated a two-dimensional structure. Confirmatory factor analysis (CFA) results confirmed that the two-factor construct demonstrated a better fit with improved psychometric characteristics. Six items were eliminated from the Thai RIBS version inventory: five items during explanatory factor analysis (EFA) and one during the CFA process. Results will contribute to ascertaining that the Thai version of the RIBS instrument can be used as a self-assessment tool for measuring students’ creative ideation. Implications and limitations of this research are discussed with suggestions for future studies.

## Introduction

One of the four competencies needed in the twenty-first century is creative thinking (P21 2019). Creative thinking is considered a high priority in both academic as well as policy-maker agendas (Ritter and Mostert 2017) and attracts interest in conducting research from many different perspectives (Tep et al. 2018). Creativity is recognized as a complicated construct that commands a vague role in educational policies (Kupers et al. 2018) but is now receiving increased attention in scholastic settings (Diakidoy and Constantinou 2001). Creativity is a multidimensional construct. This claim was first introduced by Guilford (1956) in his model of the structure of intellect. Supporting this notion, Plucker et al. (2004) conducted a content analysis of 90 articles from high impact factor journals to define the term creativity. They posited that “Creativity is the interaction among aptitude, process, and the environment by which an individual or group produces a perceptible product that is both novel and useful as defined within a social context” (p. 90). Undoubtedly, creativity involves many different aspects, and it seems entirely implausible to expect that one single all-purpose instrument can adequately assess a person’s creativity (Treffinger 2009). Several different approaches were developed to measure creativity. These included the divergent thinking test as the Torrance Tests of Creative Thinking (TTCT; Torrance 1962, 1974, 1990) and the Consensual Assessment Technique (CAT; Amabile 1982). However, these assessments are time-consuming, while the CAT is costly and requires the recruitment of experts (Baer 2016a).

Another approach to assess creativity is by using a questionnaire instrument that includes self-report indexes of creativity. However, this avenue has received considerable criticism, with questioned validity of the scales (Baer 2016b). Many researchers measured other wide-ranging aspects of creativity using a questionnaire as the simplest feasible and easy to conduct method (for a list of creativity assessment questionnaires in various aspects, see Long and Plucker 2015, p. 321). Notably, to assess creative behavioral aspects, researchers believe that creative individuals’ behavior and past experiences determine their later creativity (Colangelo et al. 1992). Conforming to this perception, many psychometric scales were developed including the Creativity Behavior Inventory (Hocevar 1979), Creative Achievement Questionnaire (Carson et al. 2005), and the Kaufman Domains of Creativity Scale (Kaufman 2012). Among all these instruments, a self-report survey as the Runco Ideational Behavior Scale (RIBS; Runco et al. 2001) was constructed to assess individual ideational behavior as an interchangeable term for thinking disposition or creative ideation. As mentioned above, creativity is often measured by different tests based on producing fluency, originality, and flexibility of ideas (Kim 2011). Idea generation precisely plays a crucial role in assessing creativity which relies on the central concept in the notion that “ideas can be treated as the products of original, divergent, and even creative thinking” (Runco et al. 2001, p. 394). Seen through the lens of the model of creativity, the RIBS can be viewed through the Four Cs model proposed by Kaufman and Beghetto (2009). According to Kaufman and Beghetto (2009), this model incorporates four aspects to measure creativity, viz., mini-c, little-c, pro-c, and big-C. At the mini-c level, creativity is measured by self-assessments whereas, at the little-c level, it is measured by additional assessments beyond self-ratings. At the pro-c level, professional’s accomplishments might be employed, and major prizes/honors are used at the big-C level (Kaufman and Beghetto 2009). Runco et al. (2001) developed the RIBS with the intention that it could be used to assess everyday creativity, despite the fact that everyone is able to produce their own ideas. Therefore, the RIBS certainly would tend to fall into the mini-c aspect. Correspondingly, Kaufman, and Beghetto (2009) indicated that “The primary purpose for assessment at the mini-c level would be to support creative ideation and nurture student creativity” (p. 8). In sum, the RIBS strongly emphasizes ideas as a product and can be used by all people in their daily lives.

Over the past two decades, RIBS has been extensively employed in numerous studies to measure different constructs, e.g., everyday creativity (Cohen and Ferrari 2010; Benedek et al. 2012b; Benedek et al. 2012a), creative ideation (Pannells and Claxton 2008; Hao et al. 2016; Tyagi et al. 2017), creative behavior (An et al. 2016; Smith et al. 2016), and ideational behavior (Batey et al. 2010; Paek and Runco 2018). It was also applied to different age groups, including children, adolescents, and elders in various studies. Sen (2016) sought to explore the latent class structure of the RIBS with 765 Turkish middle school students. The author identified three classes, i.e., regular ideators, idea-producers, and idea-averters class. Anderson et al. (2017) examined creative ideational behaviors of US 6th-grade students in relation to student engagement by using RIBS-C (RIBS for students). They found that flexibility in creative ideation was highly correlated with relational support (e.g., peers and teachers, and educational aspiration). Liu et al. (2017) assessed the mediating role of creative self-efficacy (CSE) in the relationship between active procrastination and creative ideation among the adolescents’ age group of 853 undergraduate students and found that active procrastination, CSE, and creative ideation were positively associated with each other. Pertaining to adolescents, and elder age groups, the RIBS was utilized in a study conducted by Benedek et al. (2013) along with other measurements. The authors investigated the way in which reliability and validity of the originality and fluency scores rely on subjective top-scoring method among 105 participants whose ages ranged from 18 to 51 years. Their results indicated that the “subjective top-scoring method avoids the confounding of originality scores with fluency” (p. 346). Simultaneously, many studies have investigated the underlying factor structure of RIBS, with results suggesting various solutions as one factor (Runco et al. 2001), two factors (Rojas and Tyler 2018), and three factors (von Stumm et al. 2011). Nevertheless, there are conflicting results regarding the factor structures adopted in previous studies, and also a limited number of studies related to RIBS psychometric assessment. Table 1 summarizes the published literature focusing on RIBS psychometric assessment in different languages around the world and highlights the contribution of this research in terms of adapting RIBS to the Thai language for the new location of Southeast Asia, with a large sample size compared to previous studies.

**Table 1:** Studies related to RIBS psychometric assessment in different languages

Study	Language	Location	Participants	Data analysis	Result
López-Fernández et al. (2019)	Spanish	Spain	116 not specified	Procrustes analysis	Two-factor construct
Tsai (2015)	Chinese	Taiwan	107 elementary students	EFA, CFA	Two -factor construct
Kālis and Roķe (2011)	Latvian	Latvia	107 master degree students and teachers	EFA, CFA	Two -factor construct
Runco et al. (2001)	American English	USA	224 undergraduate students	EFA, CFA	Two -factor construct (lack of theoretical support, suggesting one-factor structure)
This study	Thai	Thailand	508 undergraduate students	EFA, CFA	

*EFA* exploratory factor analysis, *CFA* confirmatory factor analysis

The RIBS psychometric has been translated into many different languages. Kālis and Roķe (2011) adapted the RIBS-09 version (49 items) to the Latvian language and administered the result to 107 Master degree psychology students and teachers. Results showed that the adapted version presented high internal consistency. Tsai (2015) also undertook a study on RIBS by adapting the original version by consisting of 23 items to the Chinese language. Tsai administered the test to 107 children in Taiwan, with results suggesting that it evinced good internal consistency among all items. Tsai further conducted a confirmatory factor analysis, and results suggested that a two-factor solution model gave a reasonable fit for the adapted Chinese RIBS instrument. Recently, López-Fernández et al. (2019) adapted the original 23-item RIBS version conducting a translation of RIBS items into Spanish. They administered the test to 116 Spanish-speaking individuals from different cities. Their results also demonstrated that two latent constructs were the best fit for the adapted Spanish RIBS. This study differs from those reported in the literature review and was conducted in a different language with a larger sample. The primary purpose of this paper was to assess the 23 items of the original RIBS in the context of the Thai language and examine scale reliability and validity (Appendix).

## Method

### Participants

To gain the maximum benefit from professional networks and ensure a high response rate from data collection, this study used a convenient sampling method in which participants were volunteers. Data were collected from undergraduate students studying in different departments of Accounting, Management, Marketing, Animal Science, Information Technology, and Educational Communication and Technology enrolled in five public universities in Thailand. A total of 524 responses were completed. Sixteen responses were excluded from the data analysis due to outliers (using a critical point of 49.73 with the Mahalanobis distance method). The final sample for data analysis comprised 508 students, 406 (80%) females, and 102 (20%) males with a mean age of 20.64 years and standard deviation of 1.27. The number of female samples in this study was more than males due to Thailand’s feminine society. In the study sample, 14.5% of the participants were freshmen, 44.1% sophomore, 21.2% juniors, and 20.2% seniors.

### Instrument

Before adapting the RIBS, the authors sought permission from Runco et al. (2001), which was granted. The questionnaire comprised two parts. The first part collected demographic information including gender, age, year levels, and subject areas. The second part focused on the original 23 self-reporting items of the RIBS that examined differences in idea usage, idea appreciation, and idea-generating skills of individuals from their usual behavior, without covert activities or actions. Participants responded using a 5-point Likert-type scale ranging from “1” being “never” and “5” being “very often.” The internal consistency of the RIBS items was highly satisfactory with Cronbach’s *α* = .94.

### Procedure

Approval and informed consent were granted by the University Institute Research Board (IRB) to carry out the study. Data collection was in accordance with human subjects’ guidelines and principles. A paper-and-pencil survey questionnaire was distributed to the participants. Before completing the questionnaire, participants were informed that their answers would remain anonymous and confidential. Participation in this survey was regarded as voluntary; participants did not gain any educational benefit (e.g., extra credit, course requirement fulfillment). This information was also written on the survey. The questionnaire was distributed to the participants and handed back in blank envelopes. In accordance with prospective participants’ discretion, they either completed the questionnaire or returned a blank or partially completed question sheet. The questionnaire items were first translated into English and then back-translated into Thai, adopting the translation-back-translation procedure of Brislin (1980). The results were verified and validated.

### Data analysis

Data analysis was conducted in three steps. In the first step, data underwent preliminary analysis to assess for missing and normality assumptions (i.e., multivariate and univariate). Notably, missing data and violation of normality assumptions did not occur in the analysis. Skewness and kurtosis ranged from − .54 to .31 and Pearson’s correlation between all items varied from .66 to .11 as less than .80 (see Table 2). In the second step, data were analyzed using exploratory factor analysis (EFA) to determine a plausible RIBS instrument factor structure. Principal axis factoring (PAF) was performed using the oblique rotation method (Promax). We employed PAF to address the number and nature of the underlying factors based on participants’ responses (Hatcher 1994) and used oblique rotation to assess the theoretically expected factor correlation. The Kaiser-Meyer-Olkin (KMO) measure confirmed whether the sample size was appropriate for EFA and CFA analyses; KMO = .95 and the KMO value of each item was > .89, higher than .5 that was specified as a satisfactory value. The value for the chi-square of Bartlett’s test of sphericity was 6,034.87, with degrees freedom at 253 and *p* < .001, suggesting that correlations between items were substantial to run PAF. A parallel analysis and scree plot were run to ascertain the number of factors to extract. All factors were selected with eigenvalues greater than 1 (Kaiser 1960). Based on Stevens (2002), the significance of loading indicated minor importance for a variable to a factor. In this regard, only factor loadings with a value higher than .4 were interpreted. The calculation of Cronbach’s alpha (α) was performed separately for each subscale to ensure measurement scale reliability.

**Table 2:** Mean, standard deviation, skewness, kurtosis, and intercorrelation between all items (*N* = 508)

Item	1	2	3	4	5	6	7	8	9	10	11	12	13	14	15	16	17	18	19	20	21	22	23
IB01																							
IB02	.60																						
IB03	.53	.44																					
IB04	.51	.49	.63																				
IB05	.52	.57	.46	.58																			
IB06	.47	.43	.60	.56	.53																		
IB07	.45	.46	.47	.54	.52	.57																	
IB08	.37	.44	.38	.48	.54	.46	.52																
IB09	.43	.48	.42	.50	.55	.49	.49	.64															
IB10	.30	.21	.45	.47	.30	.51	.44	.26	.32														
IB11	.31	.28	.28	.34	.39	.31	.33	.30	.31	.37													
IB12	.32	.33	.31	.40	.48	.38	.45	.48	.45	.33	.45												
IB13	.27	.27	.34	.37	.35	.40	.40	.38	.38	.33	.29	.39											
IB14	.27	.25	.32	.33	.40	.40	.35	.34	.30	.31	.35	.44	.44										
IB15	.31	.32	.34	.36	.45	.32	.34	.45	.38	.13	.29	.42	.35	.51									
IB16	.11	.18	.15	.20	.27	.19	.20	.26	.21	.11	.31	.28	.20	.44	.48								
IB17	.43	.41	.43	.46	.58	.45	.48	.48	.49	.34	.41	.48	.42	.51	.53	.46							
IB18	.27	.26	.26	.29	.35	.28	.30	.28	.25	.18	.38	.35	.27	.40	.45	.48	.49						
IB19	.28	.31	.43	.49	.40	.48	.44	.41	.42	.45	.30	.42	.45	.31	.36	.26	.46	.34					
IB20	.38	.32	.42	.46	.44	.42	.45	.46	.43	.32	.30	.45	.37	.39	.43	.35	.52	.43	.59				
IB21	.40	.39	.39	.50	.54	.42	.46	.43	.46	.28	.33	.44	.39	.40	.44	.28	.57	.40	.45	.62			
IB22	.36	.40	.35	.45	.47	.40	.41	.43	.41	.35	.32	.39	.40	.36	.36	.31	.50	.32	.43	.54	.59		
IB23	.44	.40	.41	.45	.54	.44	.42	.48	.51	.33	.38	.48	.42	.39	.47	.28	.57	.35	.46	.53	.66	.64	
Mean	2.88	2.70	3.17	3.1	2.75	3.21	3.11	2.69	2.75	3.64	2.88	2.72	3.01	2.81	2.64	2.62	2.77	2.67	3.05	2.89	2.8	2.85	2.75
SD	.75	.80	.96	.88	.90	.98	.95	.89	.87	1.0	.95	.92	.91	1.0	1.07	.94	.91	.97	.80	.88	.88	.93	.93
Skewness	.31	.26	− .03	.06	.13	− .11	.07	.13	.17	− .41	.16	.08	.19	.05	.28	.20	.12	.24	.02	.17	.20	.28	.31
Kurtosis	.29	.30	− .54	− .41	− .15	− .36	− .25	− . 07	− .02	− .49	− .20	− .36	− .39	− .43	− .53	− .06	− .27	− .23	− .17	− .07	− .17	− .27	− .24

In the final step, the sampling data were examined by confirmatory factor analysis (CFA) to check whether the results were well-fitted with the identified model suggested by EFA. The model fit assessment relied on several indices including the chi-square, with significant *p* value expected divided by the degrees of freedom (*χ*^2^/df), comparative fit index (CFI), Tucker-Lewis index (TLI), the standardized root mean square residual (SRMR), and the root mean square error of approximation (RMSEA). Hair et al. (2019) suggested that in the case of models that consisted of observed variables between 12 and 30 (as presented in this study), a sample number higher than 250, *χ*^2^/df < 3, CFI or TLI > .94, SRMR = .08 or less (with CFI above .94), and RMSEA < .07 with CFI of .94 or higher could be considered as a good model fit. They further suggested that using three to four indices was enough to prove the model fit.

## Results

### Exploratory factor analysis (EFA)

Results from the parallel analysis and scree plot suggested that the two-factor solution was ideal, due to cutoff point (factor loadings below .4) and cross-loading. However, five items (IB11, 12, 13, 20, and 21), e.g., “I would take a university course which was based on original ideas,” were eliminated from the Thai RIBS version inventory. The two-factor model was tested further with CFA. All items in each factor showed loading values over 0.4 (see Table 3). These two factors accounted for 48% variance: factor 1 (13 items, e.g., “I come up with a lot of ideas or solutions to problems”) and factor 2 (5 items, e.g., “I often have trouble sleeping at night, because so many ideas keep popping into my head”). The first and second factor subscales of Cronbach’s alpha values were .91 and .82, respectively. Kline (2015) suggested that good measurement models should demonstrate factor correlations that were not higher than .85. The Pearson correlation between RIBS subscales was *r* = .65, *p* < .001.

**Table 3:** Rotated component matrix of Thai RIBS version

Promax rotated factor loadings		
Item	Factor 1	Factor 2
IB04	**.83**	− .08
IB06	**.79**	− .07
IB03	**.78**	− .12
IB01	**.72**	− .09
IB07	**.71**	.01
IB02	**.65**	− .01
IB09	**.64**	.08
IB05	**.63**	.18
IB10	**.61**	− .11
IB08	**.53**	.20
IB19	**.48**	.18
IB23	**.44**	.33
IB22	**.41**	.29
IB16	− .30	**.87**
IB15	.03	**.71**
IB18	− .03	**.65**
IB14	.09	**.58**
IB17	.29	**.57**
Eigenvalues	5.77	2.86
% of variance	32	16
Cronbach’s α	.91	.82

Factor loadings over .40 are in bold

### Confirmatory factor analysis (CFA)

**Fig. 1:**
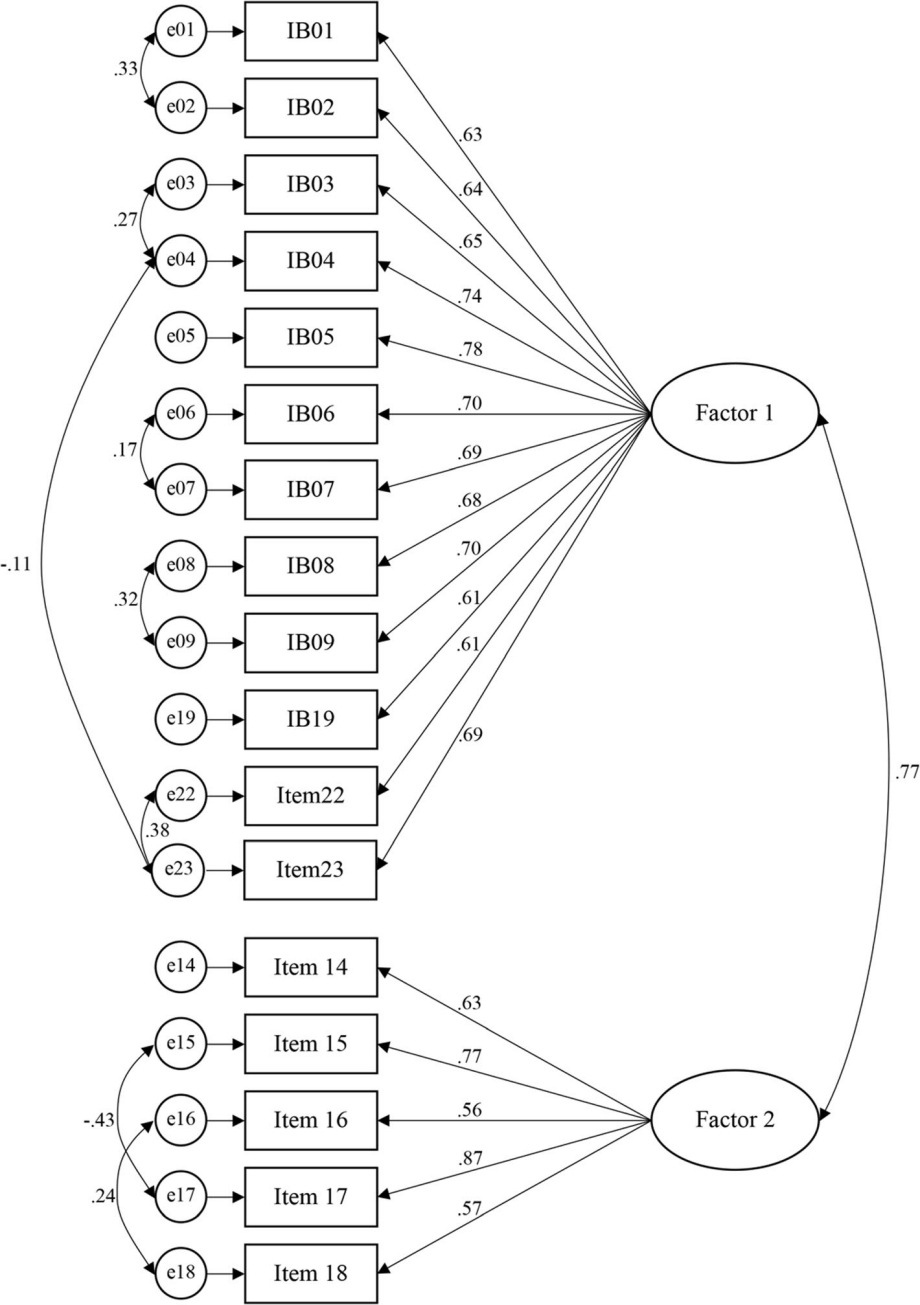
Modified two-factor model of the standardized estimates path diagram. CFA model-fit: *χ*^2^ (110) = 310.8, CFI = .95, RMSEA = 0.04, SRMR = 0.06

Diagnostics for the model noted no concerns with influential cases and assumption testing, e.g., multivariate normality. According to Runco et al. (2001), a controversy existed in choosing factor solutions. Based on the statistical notion, data were fitted for the two-factor model; however, the lack of theoretical justification for interpretation suggested that a one-factor solution was more recommendable and interpretable for the data. Therefore, differences between both constructs of one factor and two-factors were compared. As shown in Table 4, the two-construct model fitted the sampling dataset better than the single-construct model. Consequently, further modifications of the two-factor model were carried out. The modified model provided a significant fit to the sampling data. However, one item (IB10, “I enjoy having leeway in the things I do and room to make up my own mind.”) was eliminated due to nonsignificant loading (standardized factor loadings below .5). The standardized factor loadings and reliability can be seen in Table 5. The model fit indexes yielded satisfactory results and suggested that the factor structure was plausible: *χ*^2^/df = 2.82 < 3, CFI = .95 > .94, SRMR = .04 < .08, and RMSEA = .06 < .07. The path diagram of standardized estimates for a modified two-factor model is illustrated in Fig. 1.

**Table 4:** Model fit indices

Model	df	*χ*^2^	*χ*^2^/df	CFI	SRMR	RMSEA
1-factor (23 items)	230	1331.6***	5.78	.81	.06	.09
2-factor (18 items; 5 items dropped)	134	706.3***	5.27	.87	.06	.09
2-factor Modified (17 items; 6 items dropped)	110	310.8***	2.82	.95	.04	.06

*CFI* comparative fit index, *SRMR* standardized root mean square residual, *RMSEA* root mean square error of approximation

***p < .001

**Table 5:** Modified two-factor model of standardized factor loadings and reliability

Item	Factor 1	Factor 2
IB01	.63	
IB02	.64	
IB03	.65	
IB04	.74	
IB05	.78	
IB06	.71	
IB07	.69	
IB08	.68	
IB09	.70	
IB19	.61	
IB22	.61	
IB23	.69	
IB14		.63
IB15		.77
IB16		.56
IB17		.86
CR	.91	.81
AVE	.47	.48
Cronbach’s α	.91	.82

CFA model-fit: *χ*^2^ (110) = 310.8, CFI = .95, RMSEA = 0.04, SRMR = 0.06; *CR* composite reliability, *AVE* average variance extracted

Moreover, the reliabilities of both constructs of the modified model were assessed by examining convergent and discriminant validity. In terms of composite reliability (CR), CFA statistics showed that factor 1 CR = .91 and factor 2 CR = .81. Convergent validity as average variance extracted (AVE) of factor 1 was .46, with factor 2 at .47. This indicated that the CR scores of the two factors were higher than the AVE score. Therefore, this analysis demonstrated acceptable convergent validity (Hair et al. 2019). To check whether the two constructs were not different from others, the square root of AVE of both factors was calculated. The results suggested that the square root of AVE factor 1 was .67, and factor 2 was .68, respectively, which were smaller than both factor correlation value of .77. Therefore, the two constructs were related (Hair et al. 2019).

## Discussion

The original 23 items of the RIBS were adapted in the context of the Thai language to assess the structure and psychometric properties. Overall, based on the evaluation results, the Thai version presented a valid measure to a certain extent. Factor analysis suggested a two-dimensional structure. However, Tsai (2015) performed an analysis with children and conducted an EFA with results suggesting a four-factor dimension. This was likely due to the maturity and cross-cultural nature of the participants. The Thai version of the RIBS was proved to have satisfactory reliability. A one-dimensional structure was suggested by Runco et al. (2001), and a single-factor solution was called into question by Tsai (2015) and Kālis and Roķe (2011) since no theoretical support for CFA was performed regarding the fitting comparison and evaluation of this single-factor and the proposed two-factor models. Confirmatory factor analysis results confirmed that the two-factor construct demonstrated a better fit with improved psychometric characteristics. Therefore, the two-factor model was further modified for the purpose of construct validity. The revised model provided a suitable description of the data as an adequate and valid measure of ideational behavior. This result was consistent with Runco et al. (2001), Kālis and Roķe (2011), Tsai (2015), and López-Fernández et al. (2019) who all uncovered an ideal better fit two-factor construct. von Stumm et al. (2011) conducted EFA on RIBS and found that RIBS consisted of three factors as the quantities of ideas, absorption, and originality, but they did not further confirm the factor structure through CFA.

This study showed two factors as a similar result to Tsai (2015); however, item loadings into the two constructs were different. For example, Tsai’s second factor comprised items (1) I have many wild ideas, (6) I like to play around with ideas for the fun of it, (7) It is important to be able to think of bizarre and wild possibilities, (11) My ideas are often considered “impractical” or even “wild,” and (18) Some people might think me scatterbrained or absentminded because I think about a variety of things at once, whereas this study suggested that the second factor consisted of items (14) Sometimes I get so interested in a new idea that I forget about other things that I should be doing, (15) I often have trouble sleeping at night, because so many ideas keep popping into my head, (16) When writing papers or talking to people, I often have trouble staying with one topic because I think of so many things to write or say, (17) I often find that one of my ideas has led me to other ideas that have led me to other ideas, and I end up with an idea and do not know where it came from, and (18) Some people might think me scatterbrained or absentminded because I think about a variety of things at once. Tsai (2015) argued that this was due to differences between perceptions of ideational behavior and divergent thinking of Eastern and Western Cultures. Nonetheless, this study showed discrepancies with Tsai and supported the notion that there is no difference in individual innate nature of creativity as proposed by Lim and Plucker (2001).

Compared to the original study of Runco et al. (2001), the findings in this study presented the same item loadings into both factors, except that six items were eliminated from the Thai RIBS version instrument as IB10 (e.g., I enjoy having leeway in the things I do and room to make up my own mind), IB12 (e.g., I would take a university course which was based on original ideas), IB13 (e.g., I am able to think about things intensely for many hours), IB20 (e.g., I am able to think up answers to problems that haven’t already been figured out), and IB21 (e.g., I am good at combining ideas in ways that others have not tried) from construct 1 and one item (IB11, e.g., my ideas are often considered “impractical” or even “wild”) from construct 2 due to conceptual and empirical justifications. For empirical reasons, the items were dropped to improve the model validity. For conceptual reasons, all dropped items were likely related to autonomy and originality of ideas, e.g., producing new, wild ideas and combining ideas. Buasuwan (2018) suggested that Thai students’ presenting original idea behavior was likely obstructed by norms and traditions as a cultural factor that paid seniority, educators, and higher authority great respect. All item loadings in factor 1 demonstrated the number of ideas one possesses, while all items in factor 2 illustrated the barriers interrupting one’s thinking process. In order to label these two factors, this study may not have identified, more studies with theoretical interpretation are needed.

With the calling into question of the single-factor solution by Runco et al. (2001), Kālis and Roķe (2011), and Tsai (2015), this study provided clear and valid evidence that the two-factor solution could be precisely interpreted. This was due to the constructs’ convergent and discriminant validity. The Pearson correlation between RIBS subscales was also robust which smaller than .85 (Kline 2015). These findings concurred with López-Fernández et al. (2019) who indicated two explicit constructs measuring ideational behavior and the independence and number of items in both constructs. The current findings also seem to ascertain the fact that two distinct types of RIBS constructs were more reasonable.

## Limitations

The study results are limited due to several considerations. First, data collection relied on the self-rated method. Although the surveys were anonymous, students likely want to appear to be more creative than they are. In this regard, students may not provide accurate, honest answers that reflected what they really feel, which may result in false reports. Second, the number of female samples was more than males because of the feminine culture of Thailand. Convenience sampling was selected from only five universities and carried out in one country (Thailand). Thus, the findings were limited to a collectivistic Thai culture, which prevented any potential inferences. Future research should be conducted in individualistic countries or more masculine societies.

## Implications

In terms of practical implications for education, the Thai version of the RIBS can be of benefit in most schools and educators can gather a wide range of useful information regarding students’ levels of idea generation skills. Information pertaining to the barriers perceived by students might offer instructors some intervention strategies to reduce this burden. Teachers could also improve low levels of creative ideation in their students by applying enhancement strategies. Fostering creative thinking in children may differ from late adolescence. Children’s creativity manifests in the form of imagination play or self-expression; conversely, the creativity of late adolescence may lead to some products or solutions to problems (Mark and Nur 2012). Therefore, educators must consider this fact before employing different teaching strategies or techniques to different age levels of students to maximize their thinking skills. In the context of contemporary higher education, educators may consider implementing problem-based learning (PBL; Boud and Feletti 1998) along with collaborative learning (CL; Bruffee 1998) in the classroom. As a learning model, PBL encourages students to think creatively and actively. By confronting real problems, PBL may help students to generate many ideas (Hmelo-Silver 2004). When students face barriers that interrupt their thinking process, CL assumes the role of attempting to conciliate. By its nature, CL will not allow students who have problems to remain alone. Through collaboration, students learn from each other and listen to each other. This allows students, together with their instructors, to overcome and tackle the problems and obstacles placed in their path (Barkley et al. 2014).

## Conclusion

In sum, this study added theoretical knowledge regarding ideational behavior characteristics. Typically, individuals were likely to exhibit a typical frequency of generating ideas; however, sometimes they also perceived barriers in finding solutions. In addition, the results suggest that the Thai version of the RIBS instrument can be used as an additional self-assessment tool for measuring students’ creative ideation as a feasible, quick, and simple method to identify students’ ideation skills and discover whether they perceive any barriers that might be affecting their thinking process.

Finally, though the evidence reported in this study suggested that the RIBS Thai version instrument was valid and useful for assessing ideational behavior, some still doubted the usefulness of self-reported creativity assessment (Baer 2016b). Further studies should be conducted to evaluate the properties of this instrument in order to prove and put more weight on its validity and reliability.

## Figures and Tables

**Table 6:**
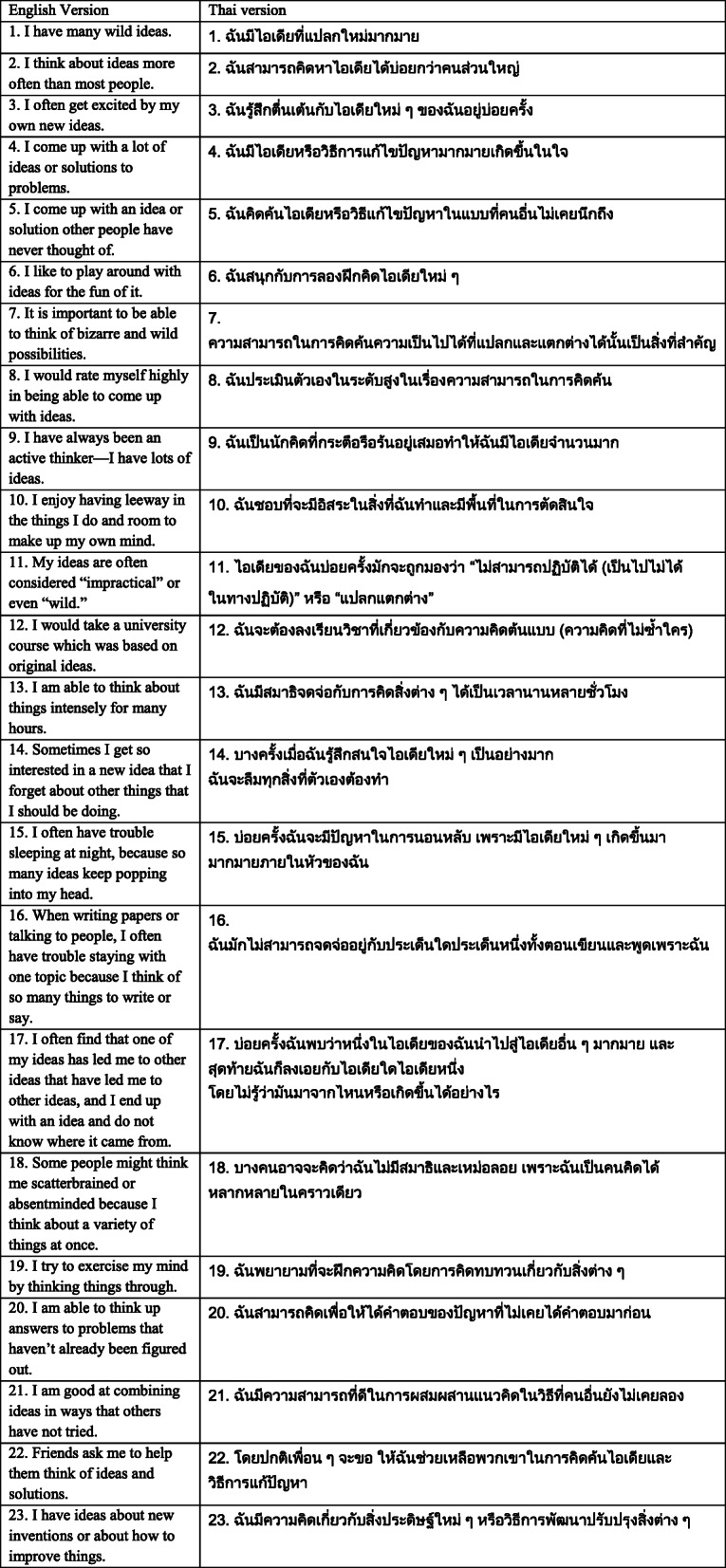
Original Runco Ideational Behavior Scale and Thai version

## Data Availability

The datasets generated during and/or analyzed during the current study are available from the corresponding author on reasonable request.

## Abbreviations

AVE: Average variance extracted
CAT: Consensual Assessment Technique
CFA: Confirmatory factor analysis
CFI: Comparative fit index
CL: Collaborative learning
CR: Composite reliability
CSE: Creative self-efficacy
DF: Degree of freedom
EFA: Exploratory factor analysis
IRB: Institute Research Board
KMO: Kaiser-Meyer-Olkin measure
PAF: Principal axis factoring
PBL: Problem-based learning
RIBS: Runco Ideational Behavior Scale
RIBS-C: Runco Ideational Behavior Scale for Students
RMSEA: Root mean square error of approximation
SRMR: Standardized root mean square residual
TLI: Tucker–Lewis Index
TTCT: Torrance Tests of Creative Thinking
